# Supplementary material for: Unistrand piRNA clusters are an evolutionarily conserved mechanism to suppress endogenous retroviruses across the Drosophila genus
Source: Nat Commun. 2023 Nov 13;14:7337. doi: 10.1038/s41467-023-42787-1 (PMC10643416; doi:10.1038/s41467-023-42787-1)
Supplement: Supplementary file 3 — Description of Additional Supplementary Files [file 41467_2023_42787_MOESM3_ESM.pdf]

Title: Supplementary Data 1:
Description: Coordinates for all unistrand flam-like piRNA cluster candidates identified in this study.

Title: Supplementary Data 2:
Description: Prediction of major de-novo piRNA clusters using somatic and total sRNA-seq data across all species sequenced in this study.

Title: Supplementary Data 3:
Description: Genomic coverage for each transposon family across all 193 assembles.

Title: Supplementary Data 4:
Description: Number of subfamilies annotated as each transposon family across all 193 assembles.

Title: Supplementary Data 5:
Description: Genomic copies per transposon family across all 193 assembles.

Title: Supplementary Data 6:
Description: Detection of 155 previously described transposon subfamilies across the curated de-novo transposon libraries.

Title: Supplementary Data 7:
Description: List of all 119 species and 193 assemblies included in this study and abbreviations used.

Title: Supplementary Data 8:
Description: Information about the fly species included in this study.

Title: Supplementary Data 9:
Description: List of all sRNA-seq libraries analysed in this study and their alignment metrics.

Title: Supplementary Data 10:
Description: List of all RNA-seq libraries analysed in this study and their alignment metrics.

Title: Supplementary Data 11:
Description: List of all ATAC-seq libraries analysed in this study and their alignment metrics
