# Supplementary material for: Unistrand piRNA clusters are an evolutionarily conserved mechanism to suppress endogenous retroviruses across the Drosophila genus
Source: Nat Commun. 2023 Nov 13;14:7337. doi: 10.1038/s41467-023-42787-1 (PMC10643416; doi:10.1038/s41467-023-42787-1)
Supplement: Supplementary file 15 — Reporting Summary [file 41467_2023_42787_MOESM15_ESM.pdf]

nature portfolio

Corresponding author(s): Susanne Bornelöv, Benjamin Czech Nicholson

Last updated by author(s): Sep 25, 2023

Reporting Summary

Nature Portfolio wishes to improve the reproducibility of the work that we publish. This form provides structure for consistency and transparency in reporting. For further information on Nature Portfolio policies, see our Editorial Policies and the Editorial Policy Checklist.

Statistics

For all statistical analyses, confirm that the following items are present in the figure legend, table legend, main text, or Methods section.

n/a	Confirmed	
☐	☒	The exact sample size (n) for each experimental group/condition, given as a discrete number and unit of measurement
☐	☒	A statement on whether measurements were taken from distinct samples or whether the same sample was measured repeatedly
☐	☒	The statistical test(s) used AND whether they are one- or two-sided<br>*Only common tests should be described solely by name; describe more complex techniques in the Methods section.*
☒	☐	A description of all covariates tested
☐	☒	A description of any assumptions or corrections, such as tests of normality and adjustment for multiple comparisons
☐	☒	A full description of the statistical parameters including central tendency (e.g. means) or other basic estimates (e.g. regression coefficient) AND variation (e.g. standard deviation) or associated estimates of uncertainty (e.g. confidence intervals)
☐	☒	For null hypothesis testing, the test statistic (e.g. F, t, r) with confidence intervals, effect sizes, degrees of freedom and P value noted<br>*Give P values as exact values whenever suitable.*
☒	☐	For Bayesian analysis, information on the choice of priors and Markov chain Monte Carlo settings
☒	☐	For hierarchical and complex designs, identification of the appropriate level for tests and full reporting of outcomes
☒	☐	Estimates of effect sizes (e.g. Cohen's d, Pearson's r), indicating how they were calculated

Our web collection on statistics for biologists contains articles on many of the points above.

Software and code

Policy information about availability of computer code

Data collection	Code to download and organise genome assemblies: https://github.com/susbo/Drosophila_unistrand_clusters/tree/main/Genome_assemblies

Data analysis	All custom scripts used in this study are available on our Github page: https://github.com/susbo/Drosophila_unistrand_clusters<br><br>Code for gene synteny analysis: https://github.com/susbo/Drosophila_unistrand_clusters/tree/main/Synteny_biogenesis_genes<br>Code to find de-novo clusters using EDTA: https://github.com/susbo/Drosophila_unistrand_clusters/tree/main/De-novo_clusters<br>Code to construct curated transposon libraries: https://github.com/susbo/Drosophila_unistrand_clusters/tree/main/Transposon_libraries<br><br>Synteny: blat v36x6, NCBI BLAST+ v2.10.0 & v2.14.0<br>Repeat annotation: RepeatMasker v4.1.2, Dfam v3.5, RepBase RepeatMaskerEdition-20181026<br>UCSC tracks: deepTools v3.3.2 & v3.5.1<br>HTS data processing: FastQC v0.11.8, Trim Galore! v0.6.4, cutadapt v1.18, bedtools v2.26.0 & v2.30.0, bowtie v1.2.3, HiSeq v2.2.0, bwa v0.7.17, SAMtools v1.9, Picard v2.9.0, ataqv v1.0.0, MACS2 v2.1.1,<br>Phylogenetic tree: treeio v1.10.0, ggtree v2.0.4, ggnewscale v0.4.6<br>Transposon libraries: EDTA v1.9.3 & v1.9.6, RepeatModeler v2.0.1 & v2.0.2a, cd-hit-est v4.8.1, full_blast https://github.com/rimjhimroy/Transposon80-80-80<br>miRNA annotations: miRBase release 22.1<br>piRNA cluster prediction: proTRAC v2.4.4<br>Assembly QC: calN50 https://github.com/lh3/calN50

For manuscripts utilizing custom algorithms or software that are central to the research but not yet described in published literature, software must be made available to editors and reviewers. We strongly encourage code deposition in a community repository (e.g. GitHub). See the Nature Portfolio guidelines for submitting code & software for further information.

Data

Policy information about availability of data

All manuscripts must include a data availability statement. This statement should provide the following information, where applicable:

- Accession codes, unique identifiers, or web links for publicly available datasets
- A description of any restrictions on data availability
- For clinical datasets or third party data, please ensure that the statement adheres to our policy

The high-throughput sequencing data and genome browser tracks generated in this study have been deposited at GEO under accession code GSE225889 [https://www.ncbi.nlm.nih.gov/geo/query/acc.cgi?acc=GSE225889]. The transposon libraries and metadata generated in this study are available on GitHub through https://github.com/susbo/Drosophila_TE_libraries. Source data are provided with this paper.

Research involving human participants, their data, or biological material

Policy information about studies with human participants or human data. See also policy information about sex, gender (identity/presentation), and sexual orientation and race, ethnicity and racism.

Reporting on sex and gender	NA
Reporting on race, ethnicity, or other socially relevant groupings	NA
Population characteristics	NA
Recruitment	NA
Ethics oversight	NA

Note that full information on the approval of the study protocol must also be provided in the manuscript.

Field-specific reporting

Please select the one below that is the best fit for your research. If you are not sure, read the appropriate sections before making your selection.

[x] Life sciences [ ] Behavioural & social sciences [ ] Ecological, evolutionary & environmental sciences

For a reference copy of the document with all sections, see nature.com/documents/nr-reporting-summary-flat.pdf

Life sciences study design

All studies must disclose on these points even when the disclosure is negative.

Sample size	No sample size calculations were done. All flies studied here were wildtype flies and therefore, no comparisons between different treatments or conditions were done. However, we sequenced at least two biological replicates for each species and assay, to ensure that there were no mistakes in the data generation.
Data exclusions	No data was excluded from our analysis. All relevant data was always incorporated and any outlier was highlighted and explained.

Replication	All sequencing data was produced in 2-4 biological replicates, in each case the replicates were successful.
Randomization	Randomization was not relevant to this study. Only wildtype flies were studied and no experimental groups were therefore present.
Blinding	Blinding was not relevant to this study. However, all sequencing libraries were processed by the same pipeline that only uses the sample annotation to select the reference genome corresponding to the species.

Reporting for specific materials, systems and methods

We require information from authors about some types of materials, experimental systems and methods used in many studies. Here, indicate whether each material, system or method listed is relevant to your study. If you are not sure if a list item applies to your research, read the appropriate section before selecting a response.

Materials & experimental systems

n/a	Involved in the study
☒	☐ Antibodies
☒	☐ Eukaryotic cell lines
☒	☐ Palaeontology and archaeology
☐	☒ Animals and other organisms
☒	☐ Clinical data
☒	☐ Dual use research of concern
☒	☐ Plants

Methods

n/a	Involved in the study
☒	☐ ChIP-seq
☒	☐ Flow cytometry
☒	☐ MRI-based neuroimaging

Animals and other research organisms

Policy information about studies involving animals; ARRIVE guidelines recommended for reporting animal research, and Sex and Gender in Research

Laboratory animals	All information on the species used in this study can also be found in Supplementary Table 8. All flies were sequenced as adults.<br><br>Species, Supplier, Strain (if known)<br>D. ananassae, Simon Collier (University of Cambridge, UK), -<br>D. azteca, National Drosophila Species Stock Center, 14012-0171.03<br>D. biarmipes, National Drosophila Species Stock Center, 14023-0361.09<br>D. bifasciata, National Drosophila Species Stock Center, 14012-0181.02<br>D. erecta, Simon Collier (University of Cambridge, UK), -<br>D. ficusphila, Geoffrey Findley (College of the holy Cross, Worcester, MA, USA)), 14025-0441.05<br>D. mojavensis, Ben Longdon (University of Exeter, UK), 15081-1352.00<br>D. persimilis, Ben Longdon (University of Exeter, UK), 14011-0111.51<br>D. pseudoobscura, Ben Longdon (University of Exeter, UK), -<br>D. simulans, David Stern (Janelia Research Campus, VA, USA), -<br>D. subobscura, National Drosophila Species Stock Center, 14011-0131.16<br>D. suzukii, National Drosophila Species Stock Center, 14023-0311.08<br>D. takahashii, National Drosophila Species Stock Center, 14022-0311.08<br>D. virilis, Simon Collier (University of Cambridge, UK), -<br>D. yakuba, Simon Collier (University of Cambridge, UK), -
Wild animals	This study did not involve any animals obtained directly from the wild.
Reporting on sex	All reported experimental research is applicable to females only. Different Drosophila species were sex sorted prior to the experiments based on their phenotype, mainly the presence of the female reproductive organ clearly visible at the posterior of the body. Only females were used for the experiments.
Field-collected samples	This study did not involve samples collected from the field.
Ethics oversight	No ethical approval or guidance was required since invertebrates and not defined as "Protected animals" in the Animals (Scientific Procedures) Act 1986.

Note that full information on the approval of the study protocol must also be provided in the manuscript.
